# Retinal Vascular Study Using OCTA in Subjects at High Genetic Risk of Developing Alzheimer’s Disease and Cardiovascular Risk Factors

**DOI:** 10.3390/jcm11113248

**Published:** 2022-06-06

**Authors:** Inés López-Cuenca, Elena Salobrar-García, Lidia Sánchez-Puebla, Eva Espejel, Lucía García del Arco, Pilar Rojas, Lorena Elvira-Hurtado, José A. Fernández-Albarral, Federico Ramírez-Toraño, Ana Barabash, Juan J. Salazar, José M. Ramírez, Rosa de Hoz, Ana I. Ramírez

**Affiliations:** 1 Ramon Castroviejo Institute of Ophthalmologic Research, Group UCM 920105, Health Research Institute of the Hospital Clínico San Carlos (IdISSC), Complutense University of Madrid, 28040 Madrid, Spain; inelopez@ucm.es (I.L.-C.); elenasalobrar@med.ucm.es (E.S.-G.); lidsan02@ucm.es (L.S.-P.); eespejel@ucm.es (E.E.); lugarc13@ucm.es (L.G.d.A.); pilar.rojas.lozano@gmail.com (P.R.); marelvir@ucm.es (L.E.-H.); joseaf08@ucm.es (J.A.F.-A.); jjsalazar@med.ucm.es (J.J.S.); ramirezs@med.ucm.es (J.M.R.); 2 Department of Immunology, Ophthalmology and ENT, Faculty of Optics and Optometry, Complutense University of Madrid, 28037 Madrid, Spain; 3 Madrid Eye Institute, Gregorio Marañón General University Hospital, 28007 Madrid, Spain; 4 Laboratory of Cognitive and Computational Neuroscience, Center for Biomedical Technology, Technical University of Madrid, 28233 Madrid, Spain; federami@ucm.es; 5 Department of Experimental Psychology, Complutense University of Madrid, 28223 Madrid, Spain; 6 Department of Endocrinology and Nutrition, Health Research Institute of the Hospital Clínico San Carlos (IdISSC), 28040 Madrid, Spain; ana.barabash@gmail.com; 7 Diabetes and Associated Metabolic Diseases Networking Biomedical Research Centre, Carlos III Health Institute, 28029 Madrid, Spain; 8 Department of Medicine II, School of Medicine, Complutense University of Madrid, 28040 Madrid, Spain; 9 Department of Immunology, Ophthalmology and ENT, School of Medicine, Complutense University of Madrid, 28040 Madrid, Spain

**Keywords:** Alzheimer’s disease, genetic risk, ApoE ɛ4, familiar history, OCTA, AngioTool, EA-Tool, retina, vascular density, cardiovascular risk factors, hypercholesterolemia, high blood pressure

## Abstract

In 103 subjects with a high genetic risk of developing Alzheimer’s disease (AD), family history (FH) of AD and ApoE ɛ4 characterization (ApoE ɛ4) were analyzed for changes in the retinal vascular network by OCTA (optical coherence tomography angiography), and AngioTool and Erlangen-Angio-Tool (EA-Tool) as imaging analysis software. Retinal vascularization was analyzed by measuring hypercholesterolemia (HCL) and high blood pressure (HBP). Angio-Tool showed a statistically significant higher percentage of area occupied by vessels in the FH+ ApoE ɛ4- group vs. in the FH+ ApoE ɛ4+ group, and EA-Tool showed statistically significant higher vascular densities in the C3 ring in the FH+ ApoE ɛ4+ group when compared with: i)FH- ApoE ɛ4- in sectors H3, H4, H10 and H11; and ii) FH+ ApoE ɛ4- in sectors H4 and H12. In participants with HCL and HBP, statistically significant changes were found, in particular using EA-Tool, both in the macular area, mainly in the deep plexus, and in the peripapillary area. In conclusion, OCTA in subjects with genetic risk factors for the development of AD showed an apparent increase in vascular density in some sectors of the retina, which was one of the first vascular changes detectable. These changes constitute a promising biomarker for monitoring the progression of pathological neuronal degeneration.

## 1. Introduction

Alzheimer’s disease (AD) is the leading cause of dementia in the world, and after patients’ advanced age, genetic heritage is one of the most important risk factors for the development of the disease. Having a parental history of Alzheimer’s increases the risk of developing the neurodegenerative disease [1,2], as does carrying at least one ɛ4 allele of apolipoprotein E (ApoE), which increases the risk of AD threefold for each allele [3,4]. ApoE ɛ4 modulates several mechanistic pathways such as cholesterol, lipid homeostasis, glucose metabolism, neurogenesis, synaptic function, Tau phosphorylation, neuroinflammation and amyloid beta (Aβ) aggregation in the central nervous system. In addition, the different ApoE genotypes modulate the function of the cerebral vasculature, reduce cerebral blood flow and increase blood–brain barrier leakage and cerebral amyloid angiopathy [5] in different ways. Research with murine models expressing ApoE ɛ4 has also identified a reduction in electroretinographic responses and lower vascular endothelial growth factor (VEGF) in the choroid and retina [6,7].

AD is characterized by the loss of brain neurons and by cerebrovascular changes in small vessels that are difficult to detect using the available brain imaging technologies [8]. Due to the similarities between the retinal and cerebral vasculature, which share embryological origin as well as physiological and anatomical properties [9], it seems logical to use the retina and its vascular network to understand the characteristics of vascular cerebral pathologies [10,11]. The retinal and cerebral vascular changes that occur in AD are caused by the accumulation of Aβ in the blood vessel wall, which leads to a decrease in blood flow that over time leads to a decrease in vascular density [12]. One hypothesis that has been proposed is a decrease in angiogenesis via the sequestration of vascular endothelial growth factor in Aβ plaques and the competitive binding of Aβ to VEGF-2 [13]. In addition, studies on APP_SWE_/PS1_ΔE_ transgenic mice have found that retinal plaques appear even earlier than in the brain and increase with disease progression [14]. Aβ deposition around vascular walls disrupts the basement membrane of small vessels, causing endothelial damage, reducing the lymphatic function of the retina and reducing the vascular lumen [13]. These pathogenic forms of Aβ can lead to the disruption of the blood–retinal barrier and to increased vascular permeability, ultimately causing neuronal damage [15]. Moreover, these changes, which are almost imperceptible using diagnostic imaging techniques, could trigger, as occurs in later stages of AD, functional changes such as those already described by Querques et al. that correspond to alterations in neurovascular coupling [16].

Optical coherence tomography angiography (OCTA) is a functional extension of OCT that allows, without the injection of any contrast, the acquisition of images that can be processed using image analysis software, enabling the retinal vascular network to be analyzed by assessing features such as the size, shape and density of the vascular blood flow [17,18,19].

Using this novel and noninvasive technology, various authors have identified changes in retinal vessels such as the loss of vascular density and increases in the foveal avascular zone (FAZ) in AD patients compared with participants with mild cognitive impairment (MCI) [20,21] and healthy individuals [22,23,24]. Vascular retinal changes have also been found in preclinical Alzheimer’s participants [25]. Furthermore, these findings indicate specific characteristics in the different stages of the disease. Thus, in subjects with mild AD, the main vascular changes occur in the choroid, and there are no vascular changes in the FAZ at this stage [26], and these findings coincide with those found in the FAZ of subjects at high genetic risk for the development of AD [27]. These results suggest that it would be of great interest to study cognitively healthy subjects because changes related to neurodegenerative disease may appear decades before the onset of cognitive symptoms [28,29], and these functional and structural features may be useful for future diagnostic procedures [1].

The aim of our work was to determine whether small morphological changes in vascular density may have already occurred in subjects with a high genetic risk of developing AD based on family history of the disease, the allelic characterization of ApoE and cardiovascular risk factors (VRF) such as hypercholesterolemia (HCL) and high blood pressure (HBP).

## 2. Materials and Methods

### 2.1. Study Desing

This work forms part of a multicenter study entitled “The cognitive and neurophysiological characteristics of subjects at high risk of developing dementia: a multidimensional approach” (COGDEM study). The ophthalmological examination was conducted at the Ramon Castroviejo Institute of Ophthalmic Research (IIORC), with the participation of other centers such as the Centre for Biomedical Technology (CBT) and the San Carlos Clinical Hospital in Madrid (HCSC). Two main groups were analyzed: participants with a family history of AD (FH+) and their controls. The (FH+) group consisted of subjects with at least one parent with sporadic AD. These subjects were not required to have a history of neurological or psychiatric disorders or to have severe disease. To ascertain the parent’s diagnosis of AD, a multidisciplinary team reviewed the parent’s medical records. All AD diagnoses were made according to internationally accepted criteria. Families with known autosomal dominant mutations (i.e., presenilin-1 or 2) were not included.

The control group (FH-) consisted of middle-aged participants with no first-degree family history of AD. This FH- group was matched with the FH+ group in terms of age, socioeconomic status and other demographic characteristics. These participants also had no history of neurological or psychiatric disorders or any serious illness. Both the FH+ and FH- groups showed normal scores (above 26) on the Mini-Mental State Examination (MMSE). All participants underwent genotyping for ApoE E4 allelic characterization.

Participants with ocular disease or posterior pole pathology, glaucoma or suspected glaucoma, a best corrected visual acuity of less than 20/40, greater than ±5 D spherocylindrical refractive error or intraocular pressure >20 mmHg were excluded from the study. The study was conducted in accordance with the tenets of the Declaration of Helsinki and was approved by the local Ethics Committee (HCSC) with the internal code 18/422-E_BS. All participants provided written informed consent.

### 2.2. Ophthalmological Study

First, the study subjects were screened by telephone. In this short interview, they were asked about their vision, such as whether they wore glasses and whether they were aware of their refractive error and approximate diopters, as well as whether they had undergone ocular surgery. All subjects who passed the telephone screening were scheduled for an appointment at the IIORC clinic for a complete ophthalmologic examination. This examination included measuring visual acuity and contrast sensitivity and analyzing color perception. The subjects also underwent a slit-lamp examination and intraocular pressure measurement with applanation tonometry, a fundus exam and an OCT and OCTA analysis.

Although both eyes of the patients were analyzed, we randomly selected one of them for the vascular analysis.

### 2.3. Classification of Patients for Retinal Vascular Network Analysis

**Figure 1:**
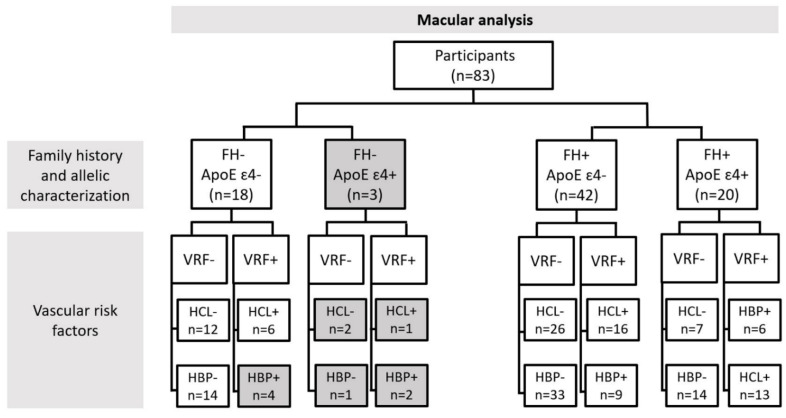
A flow diagram of the study groups according to the different characteristics for which the vascular network of the macular area was analyzed: FH: family history of AD; ApoE: Apolipoprotein E; VRF: vascular risk factors; HCL: hypercholesterolemia; HBP: high blood pressure.

For the retinal vascular network analysis, we classified the participants according to family history of AD (FH-, FH+) in addition to ApoE ɛ4 characterization (ApoE ɛ4-, ApoE ɛ4+). The first group, ApoE ɛ4-, consisted of all subjects who did not carry any ɛ4-allele, i.e., ɛ2ɛ2, ɛ2ɛ3, ɛ3ɛ3. The second group, ApoE ɛ4+, consisted of those participants who had at least one ɛ4 allele (ɛ2ɛ4, ɛ3ɛ4, ɛ4ɛ4) (Figure 1).

**Figure 2:**
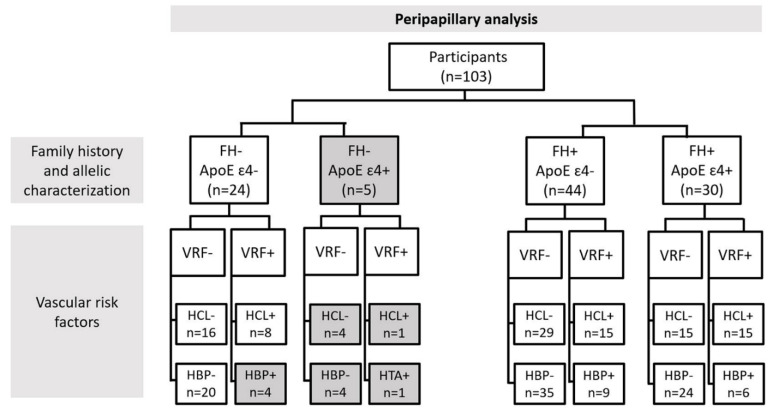
A flow diagram of the study groups according to the different characteristics for which the vascular network of the peripapillary area was analyzed: FH: family history of AD; ApoE: Apolipoprotein E; VRF: vascular risk factors; HCL: hypercholesterolemia; HBP: high blood pressure.

Finally, we analyzed the retinal vascular network by family history of AD and ApoE genotype (ApoE ɛ4- or ApoE ɛ4+), in addition to vascular risk factors (VRF-, VRF+), dividing the sample according to HCL and HBP. Figure 1 and Figure 2 show the number of participants included for the analysis of the macular area (Figure 1) and the number included for the analysis of the peripapillary area (Figure 2), following the classification explained above.

Due to this strict classification of participants, all subgroups with n < 6 subjects were discarded from the statistical analysis. Discarded groups are shown in grey in Figure 1 and Figure 2.

Only statistically significant results are shown in the tables with their respective *p*-values.

### 2.4. OCTA Acquisition and Analysis

The images were acquired using the Spectralis II OCT Angiography module (Heidelberg Engineering, Heidelberg, Germany). In our study, the macular area was analyzed using an angle of 15° × 15° and a lateral resolution of 5.7 μm/pixel. To reach the peripapillary area, the stimulus was moved to a flexible external fixation lamp and then centered on the optic nerve of the subject, and for image acquisition, an angle of 10° × 10° and resolution of 5.7 μm/pixel were used.

All images were reviewed, and any with artifacts such as being off-center, defocus, shadows, eye movements, blinking or poor segmentation were excluded. The good-quality images were exported in TIFF format for analysis using the AngioTool software and in PNG format for analysis with the EA-Tool program.

Both these vascular analysis software tools were used to analyze the vascular networks of the macular area (both the superficial and the deep plexus) and the peripapillary area (the peripapillary capillary network).

#### 2.4.1. AngioTool

**Figure 3:**
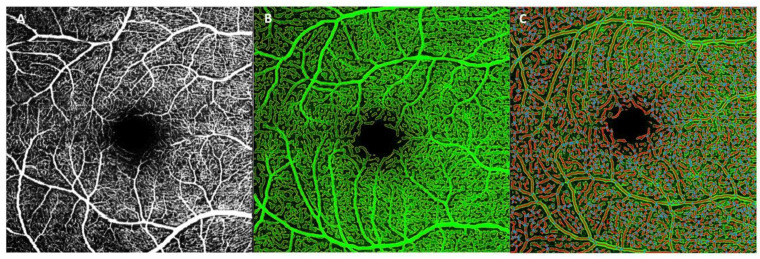
Image analysis using AngioTool software. (**A**) An OCTA image extracted from the Heidelberg image viewer in TIFF format and cut out for analysis. (**B**) The segmentation and skeletonization of vessels. (**C**) An image resulting from the analysis showing an overlay indicating the area encompassing all vessels, a skeletal representation of the vascular network and the calculated branch points within this area.

AngioTool (version 0.6a; National Institutes of Health, National Cancer Institute, Bethesda, MD, USA) was designed as a lightweight tool for angiogenesis analysis, and it enables the analysis of several vascular morphometric parameters such as vessel area, total number of junctions, junction density, average vessel length, total number of ends and lacunarity [18]. The AngioTool does not divide the retina to perform the vascular examination and analyzes the entire OCTA picture (Figure 3).

The images obtained from OCTA show the retinal vasculature in white on a dark background, making them compatible with this program. In order to analyze these images, they were extracted from the Heidelberg image viewer in TIFF format, and the analysis area was cut out (Figure 3A). The AngioTool procedure has been described by Zudaire et al. [18].

#### 2.4.2. Erlangen-AngioTool

**Figure 4:**
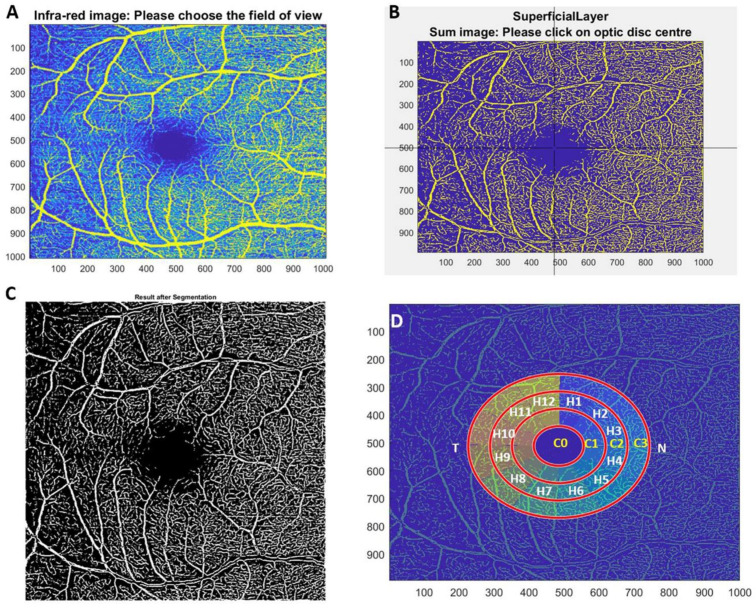
The analysis of vascular density using Erlangen-Angio-Tool software. (**A**) An infrared image where the user must delimit the analysis area. (**B**) The manual selection of the macula or optic nerve center, which is marked by a geometric grid and manually confirmed by a crosshair. (**C**) The resulting image after segmentation. (**D**) The segmentation into concentric rings and hourly sectors in which the analysis of the percentage of vascular density is performed.

Erlangen-AngioTool (EA-tool, version 1.00) is software coded in Matlab, which allows for the quantification of macular and peripapillary vessel density with reproducibility and reliability [17]. For the analysis, EA-Tool divided the OCTA into a region of interest (ROI) that was defined as 4 concentric rings (C0, C1, C2 and C3) that in turn were subdivided into 12 sectors (S1-S12) of 30° each, as described by Hosari et al. [17] (Figure 4D). For the statistical analysis of the data, the C0 ring was eliminated as it coincided in the macular area with the foveal avascular zone and in the optic nerve with the excavation and leaving of the retinal great vessels.

### 2.5. ApoE Genotyping

As indicated in previous studies conducted in our laboratory [27,30], genomic DNA was extracted from whole blood in EDTA using standard DNA isolation methods (DNAzol®; Molecular Research Center, Inc., Cincinnati, OH, USA) from all participants. Using TaqMan genotyping assays on an Applied Biosystems 7500 Fast real-time PCR instrument (Applied Biosystems, Foster City, CA, USA), two single nucleotide polymorphisms (SNPs), rs7412 and rs429358, were genotyped. In this way, the APOE haplotypes were established. Sample controls for each genotype and negative sample controls were included in each assay. Several intra- and interplate duplicates of DNA samples were also included.

### 2.6. Statistical Analysis

SPSS 27.0 (SPSS Inc., Chicago, IL, USA) was used for statistical analysis. The quantitative variables were analyzed using the Mann Whitney U test, and data are presented as median (interquartile range). A *p*-value of <0.05 was considered statistically significant.

### 2.7. Colorimetric Scale

The colorimetric scale used to represent the tables of results was created using Excel software and the color scale function. The mean values were normalized to 1, which was shown in white when there were no differences, in blue when there was a decrease and in red when there was an increase in the vascular parameters analyzed. The color scale used is shown next to each table.

## 3. Results

### 3.1. Demography

All participants included in the study were Caucasian and aged 45 to 80, and their mean cognitive score on the Mini-Mental State Examination (MMSE) was 28.81 ± 0.74.

### 3.2. Analysis of Retinal Vasculature According to Family History and the Allelic Characterization of ApoE ε4

#### 3.2.1. Macular Analysis

When we compared the macular area vascular measurements between the study groups with the AngioTool software, we found no statistically significant differences (*p* > 0.05).

The analysis using EA-Tool showed a significantly higher macular vascular density in the FH+ ApoE ɛ4- group (25.22 (21.75–27.50)) compared with the FH+ ApoE ɛ4+ group (20.65 (18.26–25.63)) in the H12 sector of the C3 ring of the deep plexus (*p*-value = 0.041) (Table 1).

#### 3.2.2. Peripapillary Analysis

The AngioTool analysis showed a statistically greater percentage area occupied by vessels in the FH+ ApoE ɛ4- group (47.52 (45.20–49.19)) compared with the FH+ ApoE ɛ4+ group (45.83(44.29–47.22); *p*-value = 0.031) (Table 1).

The EA-Tool vascular density analysis per sector identified significant changes only in the C3 ring. The comparison between FH- ApoE ɛ4- and FH+ ApoE ɛ4+ showed a significantly higher vascular density in sectors H3, H4, H10 and H11 (*p* < 0.05 in all cases) (Table 1). The comparison between FH+ ApoE ɛ4- and FH+ ApoE ɛ4+ revealed a significantly higher vascular density in sectors H4 and H12 (*p* < 0.05 in all cases) (Table 1).

### 3.3. Analysis of Retinal Vasculature According to Family History and the Allelic Characterization of ApoE ε4 in Addition to Hypercholesterolemia

None of the analyses performed with AngioTool found significant differences between the groups (*p* > 0.05).

#### 3.3.1. Macular Analysis

When the groups were analyzed using EA-Tool, we identified significant differences that are shown in Table 2. The colorimetric scale shows the normalization of the media between groups, with red and blue representing an increase and a decrease in vascular density, respectively.

When we compared FH- ApoE ɛ4- HCL- with (i) FH- ApoE ɛ4- HCL+, the second group showed statistically significantly higher vascular density in the deep vascular plexus, in the C1H11 and in the C3H9 (*p* < 0.05, in all cases); (ii) FH+ ApoE ɛ4- HCL+, we found significantly higher vascular density in the deep vascular plexus and in the C1H1, C2H1, C3H1 and C3H2 (*p* < 0.05, in all cases); (iii) FH+ ApoE ɛ4+ HCL-, we found significant vascular density changes in the superficial vascular plexus and in the C2H11 and C3H4 (*p* < 0.05, in all cases) (Table 2).

When we compared FH- ApoE ɛ4- HCL+ with: (i) FH+ ApoE ɛ4+ HCL-, the second group showed statistically significantly lower vascular density in the deep vascular plexus, in the C1H10 and in the C1H11 (*p* < 0.05, in both cases); (ii) FH+ ApoE ɛ4+ HCL+, the second group showed statistically significantly lower vascular density in the deep vascular plexus and in the C1H4 (*p* < 0.05) (Table 2).

When we compared FH+ ApoE ɛ4- HCL- with: (i) FH+ ApoE ɛ4- HCL+, the second group showed statistically significantly higher vascular density in the superficial vascular plexus; in the deep vascular plexus, and in the C1H1, C2H1, C3H2, and C3H12 (*p* < 0.05, in all cases); (ii) FH+ ApoE ɛ4+ HCL-, the second group showed statistically significantly higher superficial vascular density in the C3H4 and lower superficial density in the C1H10 (*p* < 0.05, in both cases); (iii) FH+ ApoE ɛ4+ HCL+, the second group showed statistically significantly lower deep vascular density in the C1H9 (*p* < 0.05) (Table 2).

When we compared FH+ ApoE ɛ4- HCL+ with: (i) FH+ ApoE ɛ4+ HCL-, the second group showed statistically significantly lower deep vascular density in the C1H1, C2H1, C3H11 and C3H12 (*p* < 0.05, in all cases); (ii) FH+ ApoE ɛ4+ HCL+, the second group showed statistically significantly lower superficial vascular density in deep vascular density and in the C2H1, C3H1 and C3H2 (*p* < 0.05, in all cases) (Table 2).

#### 3.3.2. Peripapillary Analysis

When we examined the vascular density around the optic nerve, we found that when we compared the FH+ ApoE ɛ4+ HCL- group with: (i) FH- ApoE ɛ4- HCL+, the second group showed statistically significantly lower vascular density in the C2H11 and C3H11 (*p* < 0.05, in both cases); (ii) FH+ ApoE ɛ4- HCL-, the second group showed statistically significantly lower vascular density in the C2H3, C3H11 and C3H12 (*p* < 0.05, in all cases) (Table 2).

When we compared FH+ ApoE ɛ4+ HCL+ with: (i) FH- ApoE ɛ4- HCL+, the second group showed statistically significant lower vascular density in the C3H3 (*p* < 0.05); (ii) FH+ ApoE ɛ4- HCL-, the second group showed statistically significantly higher vascular density in the C2H12 (*p* < 0.05) (Table 2).

### 3.4. Analysis of Retinal Vasculature According to Family History and the Allelic Characterization of ApoE ε4 in Addition to High Blood Pressure

#### 3.4.1. Macular Analysis

When we studied the vascular densities of the macular areas using the AngioTool program, statistically significant differences (decreases) were found in the total number of end points of the superficial plexus between FH+ ApoE ɛ4- HBP- and FH+ ApoE ɛ4+ HBP+ (*p* < 0.05) (Table 3).

When we used EA-Tool to compare the vascular density in FH- ApoE ɛ4- HBP- with: (i) FH+ ApoE ɛ4- HBP-, the second group showed statistically significantly higher superficial vascular density in the C1H10 (*p* < 0.05); (ii) FH+ ApoE ɛ4- HBP+, the second group showed statistically significantly lower deep vascular density in the C1H8 (*p* < 0.05); (iii) FH+ ApoE ɛ4+ HBP+, the second group showed statistically significantly higher superficial vascular density in the C1H5 (*p* < 0.05) (Table 3).

When we used EA-Tool to compare the vascular density in FH+ ApoE ɛ4- HBP- with: (i) FH+ ApoE ɛ4+ HBP-, the second group showed statistically significantly lower superficial vascular density in the deep vascular plexus and in the C1H9, C1H10, C1H11 and C1H12 (*p* < 0.05, in all instances) (Table 3).

Finally, the superficial macular vascular density in the FH+ ApoE ɛ4+ HBP+ group was significantly higher compared with FH+ ApoE ɛ4+ HBP- in the C3H11 (*p* < 0.05) (Table 3).

#### 3.4.2. Peripapillary Analysis

The AngioTool peripapillary analysis revealed significant differences between FH+ ApoE ɛ4+ HBP- and (i) FH+ ApoE ɛ4- HBP- in the % area occupied by vessels and the mean total length of vessels (*p* < 0.05, in both cases); (ii) FH+ ApoE ɛ4- HBP+ in the % area occupied by vessels (*p* < 0.05) (Table 3).

In the EA-Tool peripapillary analysis comparing the FH+ ApoE ɛ4+ HBP- group with: (i) FH+ ApoE ɛ4- HBP-, the second group showed a less significant vascular density in the C3H4 and C3H12 (*p* < 0.05, in both cases); (ii) FH+ ApoE ɛ4- HBP+, the second group showed a significantly higher vascular density in the C1H11 (*p* < 0.05) (Table 3).

In the peripapillary analysis in the FH+ ApoE ɛ4+ HBP+ group compared with: (i) FH- ApoE ɛ4- HBP-, the second group showed a less significant vascular density in the C1H11 (*p* < 0.05); (ii) FH+ ApoE ɛ4- HBP-, the second group showed a less significant vascular density in the C1H11 and C2H10 (*p* < 0.05, in both cases); (iii) FH+ ApoE ɛ4+ HBP-, the second group showed a less significant vascular density in the C1H10, C1H11 and C2H10 (*p* < 0.05, in all cases) (Table 3).

## 4. Discussion

The main finding of this study was that subjects who were cognitively healthy but had two genetic risk factors for developing AD also had a significantly higher vascular density compared with the group that had no FH and no ApoE ɛ4 allele. In addition, we found that subjects with cardiovascular risk factors also had vascular density changes in some sectors of both the peripapillary and macular areas.

Cognitive status has been seen as a vascular modifying factor in both retina and brain [31], with reports of retinal differences in both vascular dynamics and vascular and perfusion density in patients who have AD compared with subjects with MCI and healthy controls [20,32,33,34,35,36]. In addition, the trend of retinal microvasculature loss from MCI to AD may indicate retinal vascular deterioration during disease progression, contributing to the evolution from MCI to established AD [21]. The authors have also reported that the decreased velocity and blood flow of arterioles and venules coexist with the thinning of the ganglion cell-IPL complex in subjects with AD and MCI, indicating that neurodegeneration and the alteration of the hemodynamic neurovascular system overlap in these patients [34].

Furthermore, in the retina of patients with MCI, it has been found that there are already early and progressive loss of pericytes, compromised platelet-derived growth factor receptor beta (PDGFRβ) expression and the vascular accumulation of Aβ in the postmortem retina that compromise the integrity of the blood–retinal barrier [15].

In a previous work involving patients with MCI, Querques et al. [16] used the analysis of neurovascular coupling and the presence and amplitude of a typical biphasic response as a quality of autoregulation [37]. They demonstrated that changes in the vessel dynamics in MCI patients might suggest early functional alterations that precede the loss of retinal neurons. In our study sample, who were cognitively healthy participants, these increases in vascular density present in certain sectors could be the response to small functional changes occurring in neurovascular coupling before a neuronal alteration is present. Such small and localized changes go unnoticed in a general vascular density analysis as performed using the AngioTool analysis program; however, they become evident when analyzed by sector in concentric rings as performed using the EA-Tool program.

In subjects with preclinical stages of AD who present Aβ+, it has been shown that there is a statistically significantly higher vascular density compared with Aβ- subjects, both in the macular area and in the peripapillary area [38]. One possible explanation for this unexpected finding could be an inflammatory state of the retina in the early stages of amyloid accumulation as many studies have found that brain events occurring during the development of AD and Aβ accumulation are often inflammatory in nature. Assuming that these brain events occur at the same time within the retina, it may be that in the preclinical phases, this inflammatory reaction with hypoxia causes an increase in retinal blood flow. Microvessels that are not normally detected from OCTA because of low blood flow below the level of detection will become visible. This increase in the number of microvessels detected from OCTA will result in a false increased vessel density [39]. After this initial phase, continued inflammation and accumulation of Aβ can cause further damage, resulting in a loss of capillaries that in turn causes decreases in vessel density, vascular thickness and therefore flow, which explains why people with established AD have a lower vascular density [9]. This decrease in vascular density is associated with reduced angiogenesis resulting from the binding of vascular endothelial growth factor (VEGF) to Aβ and its confinement in plaque [13,40,41]. This has been demonstrated in histopathological studies in AD brains, where the accumulation of Aβ and collagen has been demonstrated within brain capillaries [42,43] that share anatomical and physiological characteristics with those of the retina [9,44]. Another study analyzing vascular changes in retinal blood vessels with respect to amyloid burden in the brain revealed changes in retinal vascular parameters such as venular asymmetry factor and arteriolar length-to-diameter ratio, with these values being higher in healthy subjects with high Aβ plaque burden compared with healthy subjects with low plaque burden. For the authors, these results indicated that changes in vascular thickness and branching occurred in early stages of the pathology, when the accumulation of Aβ deposits occurs asymptomatically, an event that precedes the cognitive deterioration of patients [31] These results support our findings that FH+ ApoE E4+ subjects showed higher vascular density in the peripapillary area in selected sectors compared with the FH- ApoE ɛ4- and FH+ ApoE ɛ4- groups.

The differences between the findings observed in the vascular network of the macular area and in the peripapillary radial capillaries, where a greater number of alterations appeared in our study, may be due to differences in their morphology. It is known that the peripapillary radial capillaries have fewer anastomoses than the capillaries of the macular superficial capillary plexus and are therefore more susceptible to vascular dysfunction [45].

However, in our study, subjects with FH+ and ApoE ɛ4+ had lower macular vascular density in the superotemporal sector (H12) of the C3 ring in the deep vascular plexus than those with FH+ and ApoE ɛ4-. This could be because our patients presented two of the main genetic risk factors for the development of neurodegenerative pathology and therefore may have already presented changes in vascular dynamics. Furthermore, these results are supported by a recent study conducted in ApoE ɛ4 carriers that found a reduction in the perfusion density in the 6 mm ETDRS macular ring and in the capillary flow index in the temporal sector compared with noncarriers. At two-year follow-up, the carriers still showed a reduction in perfusion density in the 6 mm ETDRS ring and the outer ring; however, these authors reported no differences in the rates of change between the groups [46].

Because there is a close relationship between measures of vascular function (such as perfusion pressure, arterial stiffness, carotid intima-media thickness and endothelial cell response to stress) and cognitive function [47,48], we analyzed vascular density taking into account cardiovascular risk factors such as hypertension and hypercholesterolemia, which have a strong link to reduced cerebral parenchymal blood flow and the onset of AD [49,50]. In addition, a relationship has been demonstrated between endothelial nitric oxide, which has been recognized as an important vasodilator involved in the control of vasomotor function and local blood flow [51], and cerebrovascular function, the modulation of APP processing and impairment of the functional status of microglia and of cognitive function [52].

Atherosclerosis is initiated by lipids deposited in the subendothelial layer of arterial walls that induce the expression of adhesion and chemotactic molecules. These phenomena induce the entry of monocytes/macrophages into the intima, differentiating and forming the well-known foam cells. These cells promote atherosclerotic plaque, inducing local inflammatory responses; accelerating the migration of smooth muscle cells from the intima to the media [53]; and synthesizing extracellular matrix proteins such as collagen, elastin and proteoglycans that lead to vascular remodeling and decreased flow [54]. These phenomena, which occur with the inflammation and hypoxia produced by Aβ deposition in very early stages, could cause an increase in the flow through these vessels, making them visible via OCTA, which would explain the small increase in macular vascular density observed in our patients with HCL [39].

Arterial hypertension, which causes arteriolar narrowing and venular widening in the retinal circulation, is a known risk factor for the development of AD [55]. Some studies have shown that these vascular changes may precede the development of clinical hypertension [56]. In our study, it was observed that those subjects with two genetic risk factors for the development of AD and with HBP had a higher vascular density than did those without HBP. This increase observed in OCTA may be due to the increased flow caused by vascular narrowing, which makes previously imperceptible vessels visible. Another possible explanation is that venular widening causes OCTA to detect vessels of larger caliber, resulting in higher vascular density.

This study also revealed different involvement between the deep and superficial macular vascular plexus. The superficial vascular plexus is found in the retinal nerve fiber and ganglion cell layers, whereas the deep vascular plexus irrigates the inner plexiform as well as the inner nuclear and part of the outer plexiform layers. Previous studies have reported that the deep vascular plexus is affected earlier and more severely than the superficial vascular plexus, a possible explanation being the type of vessels of which they are composed: the deep plexus is formed by capillaries, whereas the superficial plexus is composed of larger vessels such as precapillary arterioles, capillaries and postcapillary venules [21]. In this study, most of the changes were observed in the deep vascular plexus, supporting the theory that in subjects at genetic risk for the development of AD, the earliest changes in retinal thickness are in the inner plexiform layer [30]. It has also been considered that the involvement of this plexus may be related to the progression of the disease [57]. However, other studies have found no involvement of the deep vascular plexus, and it is the superficial plexus that undergoes more vascular changes; these hypotheses are supported by the involvement of the inner retinal layers (retinal fiber and ganglion cell) during the disease [36].

Our study has strengths but also limitations. This is the first study to analyze vascular density in clinically ophthalmologically healthy and cognitively healthy subjects at high genetic risk for the development of AD. In addition, the analysis of OCTA images was performed using two software tools that analyze different and complementary parameters (AngioTool and EA-Tool).

One of the aspects worth noting in relation to this study was the difference in participant numbers in the macular and peripapillary analyses. This difference was due to the strict selection of images. In our study, images with any type of artifact were discarded. Although artifacts are common in OCTA images [58], it has been shown that these could have implications for quantitative results, challenging the interpretability and reproducibility of the proposed parameters for clinical trials [59]. The other limitation is the small sample size, and studies with larger numbers of patients and longitudinal studies are needed to determine whether these vascular changes can be used in clinical practice as biomarkers of AD.

Another point of discussion in our study is the age range of the participants (from 45 to 75), but there is no consensus on whether this interferes with ocular vascular density. Although Shahlaee et al. found a negative correlation between vascular density and age [60], other authors have shown that the vascular densities of both the superficial and deep plexus are not affected by age [61].

Finally, this is one of the first exploratory studies to investigate vascular density in this type of participant in detail. In accordance with this exploratory character, we decided to take a more flexible approach to the problem of multiple comparisons. We believe that these results may serve as a first step or guide for new hypotheses and future studies that validate our results and may reveal new biomarkers for AD.

## 5. Conclusions

In conclusion, using OCTA in subjects with two genetic risk factors for the development of AD, an apparent increase in vascular density was detected in some sectors of the retina, probably due to the opening of arteriovenous shunts. These changes could be one of the first vascular changes detectable using OCTA in this type of subject. In addition, the alteration of vascular dynamics caused by HCL and HBP could be compensated by a slight increase in vascular density in the retina, which can be detected via detailed OCTA analysis by sector and a concentric ring as performed using the EA-Tool.

Measurements taken with OCTA may constitute a promising biomarker for monitoring the progression of pathological neuronal degeneration associated with AD and cardiovascular disease.

## Figures and Tables

**Table 1:** The vascular analysis of significant data according to family history and the characterization of ApoE ɛ4.

Macular analysis								
Software	Plexus	Ring	Sector	FH- ApoE ɛ4-	FH+ ApoE ɛ4-	FH+ ApoE ɛ4+	FH- ApoE ɛ4- vs. FH+ ApoE ɛ4+	FH+ ApoE ɛ4- vs. FH+ ApoE ɛ4+
				(n = 18)	(n= 42)	(n = 20)	*p*-value	
EA-Tool	DVP	C3	H12	23.3 (18.4–27.4)	25.2 (21.7–27.4)	20.6 (18.2–25.6)	0.313	0.041 *
Peripapillary analysis								
Software	Plexus	Ring	Sector	FH- ApoE ɛ4-	FH+ ApoE ɛ4-	FH+ ApoE ɛ4+	FH- ApoE ɛ4- vs. FH+ ApoE ɛ4+	FH+ ApoE ɛ4- vs. FH+ ApoE ɛ4+
				(n = 24)	(n= 44)	(n = 30)	*p*-value	
AngioTool	% Area occupied by vessels			47.1 (43.7–48.6)	47.5 (45.2–49.1)	45.8 (44.2–47.2)	0.423	0.031 *
EA-Tool	EA-Tool	C3	H3	17.2 (12.0–22.6)	16.9 (13.1–27.4)	23.2 (17.4–30.5)	0.021*	0.073
			H4	16.0 (11.3–31.3)	19.6 (13.9–31.1)	23.9 (20.3–30.7)	0.030 *	0.049 *
			H10	22.1 (17.5–26.2)	24.1 (17.6–27.9)	27.2 (20.7–31.8)	0.037 *	0.151
			H11	23.9 (17.4–27.6)	24.0 (15.1–32.0)	27.9 (23.2–37.9)	0.031 *	0.057
			H12	22.8 (14.1–32.5)	17.8 (10.6–29.6)	31.5 (16.4–39.1)	0.280	0.023 *

Median (IR); * *p* < 0.05 Mann-Whitney U test. HF: Family history; ApoE; vs: versus; EA-Tool: Earlagen Tool; DVP: Deep vascular plexus; IR: interquartile range.

**Table 2:**
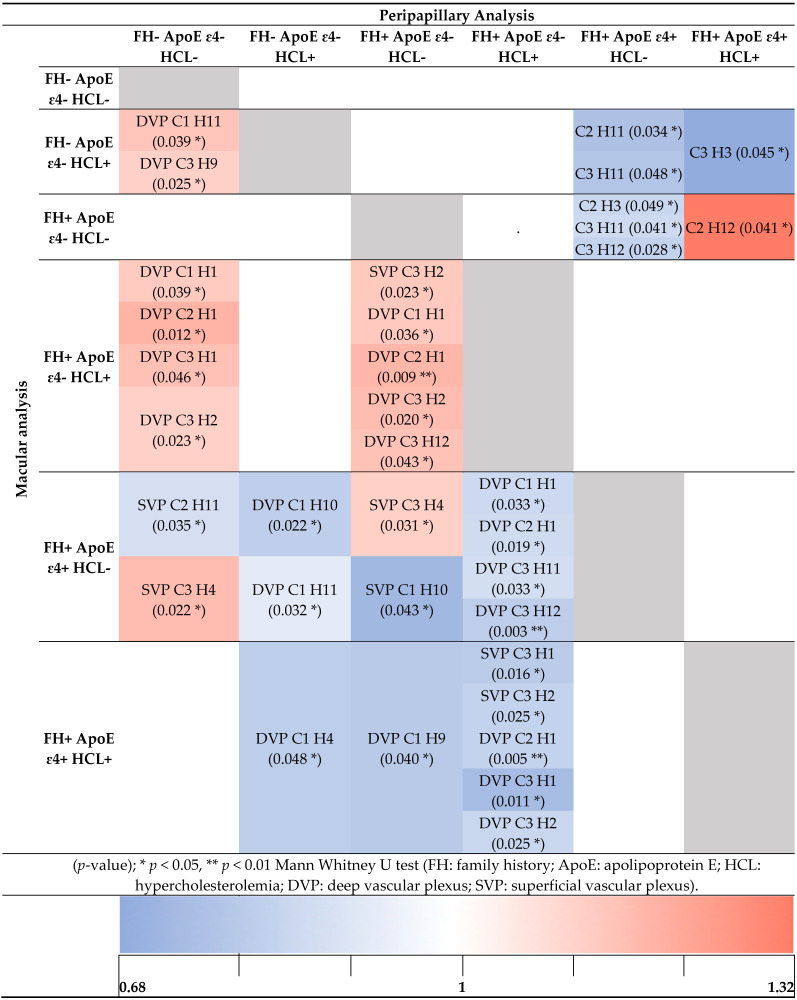
The *p* values of the macular vascular densities of the subjects classified by family history and the allelic characterization of ApoE ɛ4 and HCL using the EA-Tool.

**Table 3:**
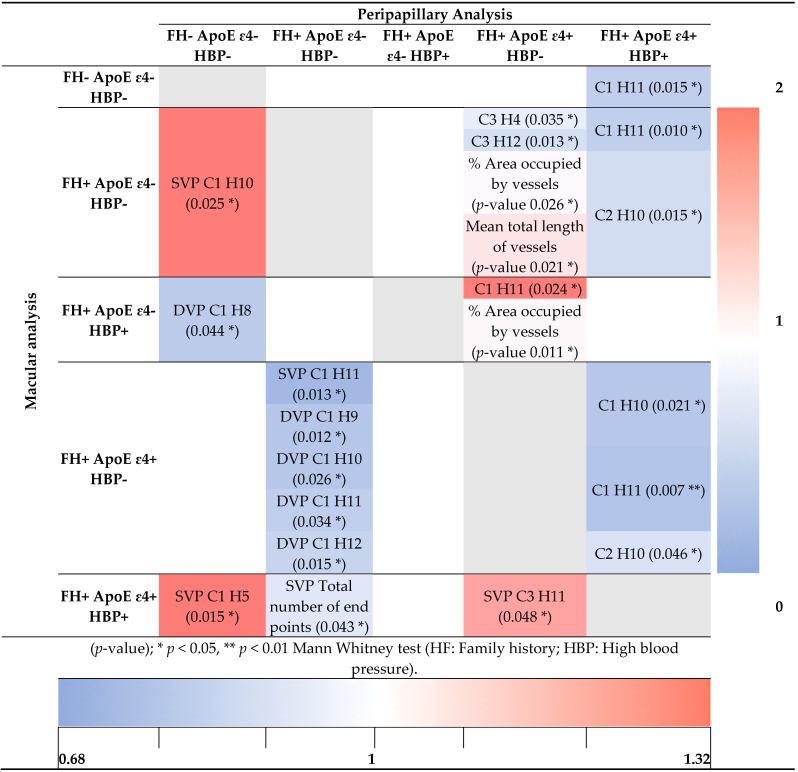
Vascular density according to family history of AD and the allelic characterization of ApoE and HBP using AngioTool and EA-Tool.

## Data Availability

The data supporting the findings of this study are available from the corresponding author upon request.
